# A real-time Global Warming Index

**DOI:** 10.1038/s41598-017-14828-5

**Published:** 2017-11-13

**Authors:** K. Haustein, M. R. Allen, P. M. Forster, F. E. L. Otto, D. M. Mitchell, H. D. Matthews, D. J. Frame

**Affiliations:** 1 Environmental Change Institute, University of Oxford, Oxford, UK; 2 Department of Physics, University of Oxford, Oxford, UK; 3 School of Earth and Environment, University of Leeds, Leeds, UK; 4 Department of Geography Planning and Environment, Concordia University Montreal, Montreal, Canada; 5 New Zealand Climate Change Research Institute, School of Geography, Environment and Earth Sciences, Victoria University of Wellington, Wellington, New Zealand; 6 Present Address: School of Geographical Sciences, University of Bristol, Bristol, United Kingdom

## Abstract

We propose a simple real-time index of global human-induced warming and assess its robustness to uncertainties in climate forcing and short-term climate fluctuations. This index provides improved scientific context for temperature stabilisation targets and has the potential to decrease the volatility of climate policy. We quantify uncertainties arising from temperature observations, climate radiative forcings, internal variability and the model response. Our index and the associated rate of human-induced warming is compatible with a range of other more sophisticated methods to estimate the human contribution to observed global temperature change.

## Introduction

2016 was the third year in a row with a record-setting global mean surface temperature (GMST), inviting the question of how much of this burst of recent warming is due to human activities, and how much to natural variability, just as the purported “lack of warming” has been scrutinized before 2014 for the opposite reason^1–9^. This issue matters for policy, given the decision made in UN negotiations to structure international aims around global temperature targets; and it matters even more in view of the proximity of the lower of the two figures mentioned in the 2015 Paris Agreement which has the stated aims of “*Holding the increase in the global average temperature to well below 2* °C *above pre*-*industrial levels and pursuing efforts to limit the temperature increase to 1*.*5* °C *above pre*-*industrial levels*”. While scientists are familiar with the role of natural variability in GMST, now and on future trajectories^10,11^, policymakers have not (to date) accounted for natural variability in UNFCCC negotiating texts. The phrase “global average temperature” could be given several reasonable interpretations, including annual observed GMST as determined by a single data series; annual observed GMST as determined by several data series; multi-year running means; or, as we argue here, assessments of the anthropogenic component of the observed warming.

Otto *et al*.^12^ proposed an index of human-induced warming based on a simple least-squares fit between observed temperatures and the expected responses to anthropogenic and natural radiative forcing^13^. However, they did not provide a thorough assessment of its robustness, its sensitivity to internal variability, or its uncertainty in natural or anthropogenic forcings. They primarily focused on illustrating the utility of such a concept in adaptive mitigation. Here we present a coherent uncertainty analysis for a real-time version of the proposed warming index that satisfies the need for immediate availability. We update it using monthly GMST and revised monthly radiative forcing data. These timely updates are important should an explosive volcanic eruption occur which will lower GMST almost instantly^14^. As a reference period we use 1850–79, which represents the earliest 30 year period in most observational records. Since some volcanic activity is recorded in the 1850s, we also test the 1861–1880 period. Years after 1880 are affected by the very negative Krakatoa forcing. Use of earlier reference periods would be closer to “pre-industrial”^15^, but that is not possible due to the absence of global instrumental data. The IPCC has often used 1851–1900 as pre-industrial reference period, prompting us to repeat the analysis one more time with this interval.

Whether or not fluctuations in GMST (such as the so-called “hiatus”)^16^ and the scientific response thereto^17^ have contributed to slow down progress of climate negotiations is a moot point. Now that we have, through the Paris Agreement, an explicit global temperature goal, we believe it is all the more necessary to have an easily accessible and understandable index of total anthropogenic warming, designed to reduce effects of internal and naturally-driven climate variability.

## Methodology and Uncertainty Analysis

A variety of detection and attribution techniques have been used to conclude consistently that most of the observed increase in near-surface temperatures over the second half of the 20th century is attributable to anthropogenic influences^13,18–21^. The Global Warming Index, or GWI, proposed here is based on the standard “multi-fingerprinting” approach introduced by Hasselmann^13^, as also used by the IPCC and, for example, by Folland *et al*.^22^ who adopted a very similar approach to estimate temperature responses. The “multi-fingerprinting” method is, in essence, a linear regression, taking observed GMST (as provided by the UK MetOffice HadCRUT4 dataset^23^ together with a variant using Kriging to infill regions without data^24^, hereinafter referred to as HadCRUT4-CW) as the dependent variable and estimated responses to human-induced and natural drivers of climate change as the independent variables.

Applying the methodology introduced in Otto *et al*.^12^, we use the most recent radiative forcing estimates based on IPCC AR5 (Chapter 8)^25^, updated to 2017 using Greenhouse Gas (GHG) data from NOAA and ECLIPSE (Evaluating the Climate and Air Quality Impacts of Short-Lived Pollutants)^26,27^, including a higher methane (CH_4_) forcing estimate^28^, ozone (O_3_) and anthropogenic aerosol data from CICERO^29^, solar variability data from the Solar Radiation and Climate Experiment (SORCE)^30^ and volcanic forcing data from NASA/GISS^31^. Radiative forcing due to land-use changes and indirect aerosol effects are included. The annual data before 2015 are interpolated to monthly values (except for volcanoes where we use monthly data throughout).

The temporal evolution of the responses to total anthropogenic (combined greenhouse gas and aerosol) and natural (combined solar and volcanic) radiative forcing is calculated using the two-time-constant IPCC impulse-response model^25,32^. The best estimate for the fast (slow) adjustment time is 4.1 (209) years^33^. Long-term slow recovery from volcanic eruptions^34–36^ is hence represented in the model. The TCR/ECS ratio (Transient Climate Response/Equilibrium Climate Sensitivity; also referred to as Realised Warming Fraction^37^) is 0.6, close to the CMIP5 model mean of 0.55. The anthropogenic contribution to climate change is determined by a simple least-squares-fit between observed temperatures and anthropogenic and natural forcing contributions^38^, allowing observed GMST to be decomposed into human-induced and natural components. Ordinary least squares is appropriate because the output of the simple model is almost free of stochastic variability, hence the noise is very small.

The 5–95% confidence interval in the GWI is estimated on the basis of observational (100 possible variations^23^), forcing (200 possible variations^25,39^), response model uncertainty (20 possible variations^33^) and uncertainty due to internal variability (50 possible variations, using internal variability from the CMIP5 pre-industrial control ensemble). Drifting control runs are rejected above a certain threshold (±0.15 °C/century). We note that the magnitude of internal variability could be overestimated with our method given that low frequency variability in CMIP5 models requires careful calibration^40^. It may even change in the future^41^. The radiative forcing time-history uncertainty is estimated using a Monte Carlo method^42,43^. The response model uncertainty covers a wide range of Realised Warming Fractions (0.2–0.8) as well as a range of fast adjustment timescales (2–10 years). This includes slower fast response times, consistent with a real world response different from GCMs^44^. Since the contribution of the impulse response model uncertainty to the overall uncertainty is small, we retain ~4 years for the fast response time.

## Results and Discussion

**Figure 1:**
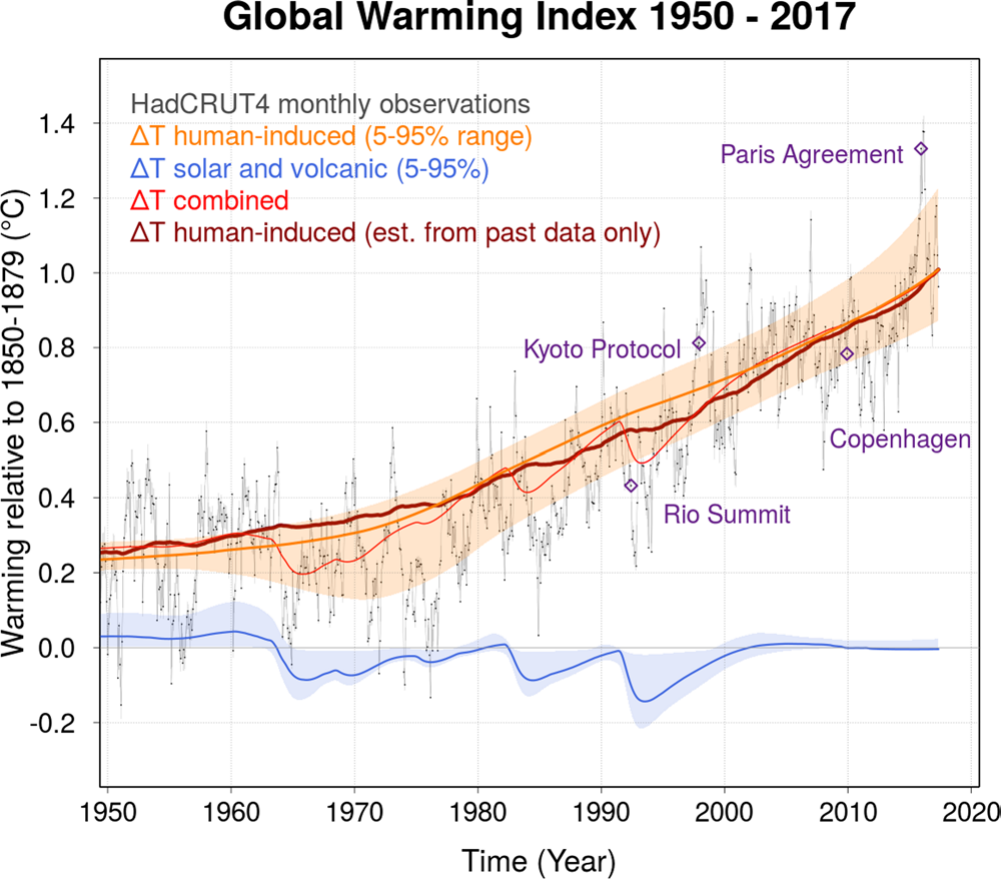
Global Warming Index from Jan 1950 to May 2017 for HadCRUT4. The anthropogenic contribution in orange (with 5–95% confidence interval). The natural contribution (solar and volcanic) in blue. The red line shows the combined (total) externally-driven temperature change. The dark red line shows the evolution of the GWI when only past forcing and temperature data are used. It starts in 1944 - the time when a human-induced warming signal can first be detected - followed by a new data point for each month up until May 2017. The evolution of the red line indicates the degree of month-to-month variability of the index. The thin black line are the monthly (HadCRUT4) GMST data. For illustration, blue diamonds indicate when major climate summits took place in context of the monthly GMST at that time.

Using HadCRUT4, the human-induced warming in May 2017, calculated relative to the period 1850–79, reached +1.01 °C with an uncertainty range of +0.87 to +1.22 °C (5–95% confidence interval) as shown in Fig. 1 (orange line). The corresponding natural externally-driven change is −0.01 ± 0.03 °C and hence very small in comparison to the human contribution (blue line in Fig. 1). Essentially all the observed warming since 1850–79 is anthropogenic. Using HadCRUT4-CW instead, the GWI for May 2017 is +1.08 °C with an uncertainty range of 0.92 to 1.32 °C (see Fig. S1b in the supplementary material).

**Figure 2:**
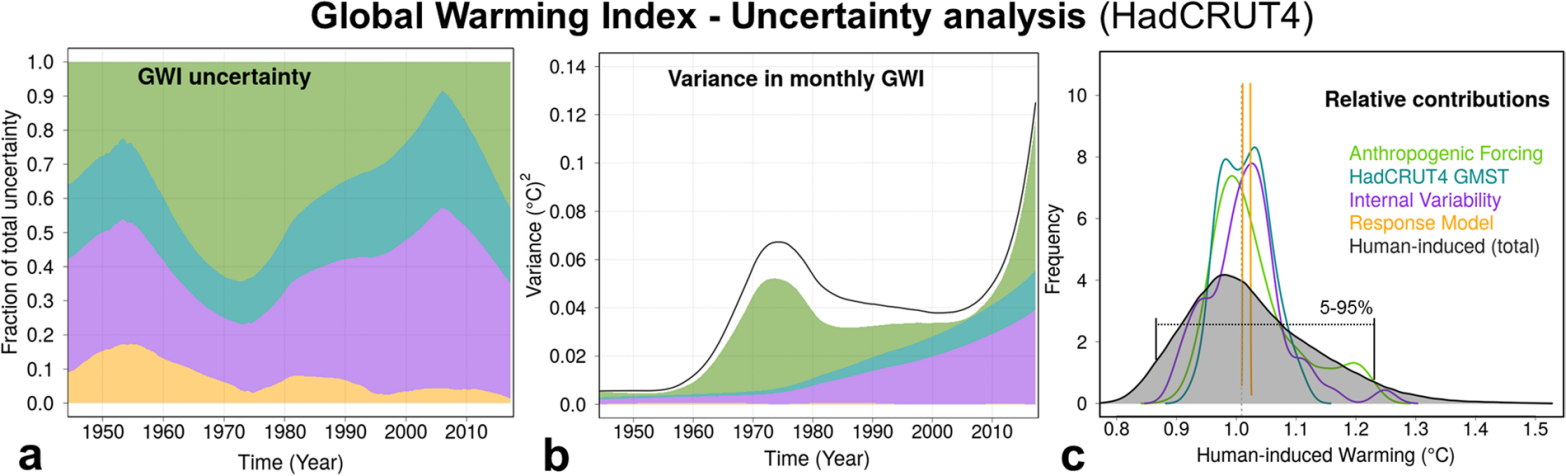
GWI uncertainty analysis for HadCRUT4. (**a**) Temporal evolution (1950–2017) of fraction contributions to the total uncertainty (5–95% range). (**b**) Same as (**a**) but for the variance (square of the standard error). The black line is the combined total variance. This differs from the sum because uncertainties are non-gaussian. Note the very small contribution of the response model uncertainty at the bottom. (**c**) Probability density function for the relative uncertainty contributions to the value of the GWI in May 2017. Note that the response model frequency peaks at ~100. In black is the PDF of the combined total uncertainty.

Our uncertainty estimates are consistent with other attribution results: We estimate human-induced warming over 1951–2010 to be +0.63 ± 0.08 °C (105% of the total warming) in HadCRUT4 (+0.65 °C for HadCRUT4-CW; Fig. S1b), in line with the 2013 IPCC assessment (given to 1 decimal place) of +0.7 ± 0.1 °C based on more comprehensive attribution studies using the CMIP5 ensemble. The contributions to the uncertainty ranges (5–95% interval) for the current level of warming are −0.06 to +0.07 °C (observational), −0.07 to +0.19 °C (climate forcing), −0.002 to +0.009 °C (response model), and −0.09 to +0.12 °C (internal variability). The fraction of the contributions to the total uncertainty, the variance and the distributions of the decomposed uncertainty estimates are shown in Fig. 2a–c, respectively. The observational uncertainty combines effects of measurement, sampling, bias, and coverage uncertainties. It is described in the HadCRUT4 uncertainty model^23,45–47^ and applied to HadCRUT4-CW in the same way (see Fig. S2 in the supplementary material). We note that the coverage uncertainty in HadCRUT4 might be underestimated as it does not account for different warming rates over unobserved or under-sampled regions such as the Arctic. The overall uncertainty is dominated by the radiative forcing uncertainty as shown in Fig. 2a,b.

Importantly, uncertainties in GWI are primarily systematic, and hence do not increase its temporal volatility. Re-computing the GWI based only on data up to different points in the past leads to variations of no more than ±0.1 °C relative to the estimate based on the full data available now (dark red compared to orange line in Fig. 1). These deviations are strongly auto-correlated and declining as the signal strengthens. Hence the GWI is relatively insensitive to the end date as well as short-term GMST fluctuations, pre-empting possible misconceptions, for example, that a strong El Niño or La Niña event represent an acceleration, “hiatus”, or slowdown in human-induced warming.

The rate of human-induced warming is estimated using the latest 20 years of data. The most recent 20 year trend is +0.16 °C/decade in HadCRUT4, and the uncertainty range spans from +0.12 to +0.32 °C/decade. For HadCRUT4-CW, the trend is +0.17 °C/decade (0.13–0.33 °C/decade). The range is skewed due to the one-sided uncertainty related to anthropogenic aerosols during 1950–80. Our warming rate estimate is compatible with the 1979–2010 trend calculated in Foster and Rahmstorf^48^ using multiple regression to filter out natural contributors (+0.17 °C for HadCRUT4). Other more physically based methods to remove natural variations yield equally compatible results^49^. Trends of shorter time intervals show larger anthropogenic warming rates in recent years, indicating that the trend in GHG forcing has likely been increasing, although CO_2_ emissions have stabilised during the last three years. Non-CO_2_ GHGs such as methane may be responsible for the accelerated anthropogenic warming rate^28^, with perhaps a minor contribution from reduced Asian aerosol pollution^29,50^.

Up until this point, we have presented results for HadCRUT4 and HadCRUT4-CW, which differ in their global coverage and hence do have a noticeable effect on our results. Since the HadCRUT4-CW method has only been fully published for the data after 1979^24^ (with a brief reference to the data before 1979 in the supplementary material of Cowtan *et al*.^51^), we use HadCRUT4 as our main result for the time being. We note, however, that HadCRUT4 has known negative biases due to incomplete coverage of the Arctic where the globe is warming the fastest^52^ which may lead to an underestimated anthropogenic warming^6^.

To further test the notion that our results are sensitive to the choice of the GMST dataset, we analysed NASA/GISS^53^, the NOAA Merged LandOcean Surface Temperature Analysis^54^ and the Berkeley Earth Surface Temperature analysis^55^ in the same way (see Fig. S3 in the supplementary material). Note that NASA/GISS and NOAA/MLOST data are only available after 1880, i.e. the reference period is 1881–1910. The GWI for May 2017 varies from +1.08 °C  (NOAA) to +1.13 °C  (Berkeley), suggesting that the current warming rate in HadCRUT4 may indeed be at the lower end. That said, NASA/GISS, for example, uses ERSSTv4^56^ over oceans, which appears to have substantial biases during WWII^57^. While attempts have been made to correct this bias in ERSSTv5^57^, the problem may still not be resolved. HadSST3 (used in HadCRUT4, HadCRUT4-CW and Berkeley Earth) does not show the same anomalous behaviour. Accordingly, our fit between total forcing and NASA/GISS is worse than for the two HadCRUT4 versions (Fig. S3c). The same is true for NOAA/MLOST (Fig. S3d). Berkeley Earth has gaps in the early part of the record and NOAA has no Arctic coverage either. Hence there really is no “perfect” GMST dataset at the moment, which is why we argue that it is defensible to use HadCRUT4. Future versions of those GMST datasets may come in to better agreement as data gaps are closed and methodologies improved.

As far as the choice of the reference period is concerned, in Fig. S1 we illustrate the effect quantitatively. While the difference between the volcano-free 1861–80 period and the 1850–79 default reference period is not substantial (−0.005 °C across the datasets), using the 1851–1900 reference period instead does have a significant effect on the main result. The index is reduced by ~0.02 °C due to a shifting mean reference time for the anthropogenic forcing to 1875. Natural forcing contributions are negative during the 1881–1900 period, moving the natural warming contribution to positive values when the 1851–1900 reference period is used. Conveniently, the current natural warming contribution is essentially zero in the default reference period of 1850–79.

Despite this array of uncertainties, the GWI comes with a number of advantages compared to using GMST from observations or GCMs as global warming metric. The most important advantage is that it does not depend on climate models to estimate the anthropogenic warming fraction: we make no prior assumptions about the magnitude of the response to either anthropogenic or natural forcing, or the climate sensitivity, in computing the GWI. Nonetheless, there are a few issues that need to be considered. While the GWI reflects global forcing responses by construction, it implicitly assumes globally homogeneous forcing. However, anthropogenic aerosols are emitted spatially very inhomogeneous. As demonstrated elsewhere^5,58^, this asymmetry leads to globally inhomogeneous temperature responses which need to be considered. More land surface area and a generally higher aerosol load over land leads to faster forcing response times in the Northern Hemisphere in comparison to the ocean-dominated, cleaner Southern Hemisphere. We therefore tested the sensitivity of hemispherically asymmetric aerosol forcing upon our results by using different response times in both hemispheres. We find that the current GWI is insensitive to the treatment of anthropogenic aerosols. This can be explained by the fairly constant aerosol emission levels over the last 10–20 years as far as the resulting total hemispheric forcing is concerned.

## Conclusions

We have proposed an operationally-updated Global Warming Index following the well established “multi-fingerprinting” approach^13^, estimating anthropogenic warming by computing an ordinary least-squares regression with observed GMST as dependent variable and the responses to anthropogenic and natural forcing as the two independent variables. For GMST, we use the UK MetOffice HadCRUT4. The infilled version (HadCRUT4-CW) is shown to provide contextual information with regard to uncertainties related to the choice of the GMST dataset. The response model to convert the forcing into the associated temperature response uses fast and slow time constants^33^.

The central estimate of current anthropogenic warming (+1.01 °C as of May 2017) is well below the monthly peak warming of +1.35 °C in Feb and Mar 2016 and well above the colder pre-2013 period. The rate of human-induced warming may actually be accelerating over the past 20 years and is currently at +0.16 °C/decade based on the 1997–2016 period. The index is insensitive to the aerosol forcing and relatively insensitive to the end date and GMST fluctuations on inter-annual or even inter-decadal timescales. For example, the GWI would have increased monotonically throughout the so-called “hiatus” period^59^. We find that there is a considerable difference between our HadCRUT4 and HadCRUT4-CW results due to the coverage issue in the Arctic. GMST warming does not tell the whole climate change story as land warms at least twice as fast as oceans under transient climate change conditions. However, the attribution question remains largely unaffected as both land and ocean show distinct warming patterns that are linked to anthropogenic forcing.

An easily accessible index (see www.globalwarmingindex.org) of how much of the observed warming is attributable to human influence is helpful information that should be permanently available to anyone. Robustness, transparency and salience of the GWI render it a reliable and usable benchmark tool. While the very robustness we have demonstrated here leads to a stable index with little to no month-to-month variability, the real-time factor is indispensable when it comes communicating to the public. Institutions such as the World Meteorological Organisation could provide a regular standardised update of human-induced warming to support future stock-takes of progress towards climate stabilisation. Attribution can no longer be left to periodic academic assessments.

## Electronic supplementary material

Supplementary Information
