# Supplementary material for: A real-time Global Warming Index
Source: Sci Rep. 2017 Nov 13;7:15417. doi: 10.1038/s41598-017-14828-5 (PMC5684339; doi:10.1038/s41598-017-14828-5)
Supplement: Supplementary file 1 — Supplementary Information [file 41598_2017_14828_MOESM1_ESM.pdf]

Supplementary Material for "A real-time Global Warming Index"

K. Haustein[1], M. R. Allen[1,2], P. M. Forster[3], F. E. L. Otto[1], D. M. Mitchell[1], H. D. Matthews[4] & D. Frame[5]

[1]*Environmental Change Institute, University of Oxford, UK*

[2]*Department of Physics, University of Oxford, UK*

[3]*School of Earth and Environment, University of Leeds, UK*

[4]*Department of Geography Planning and Environment, Concordia University Montreal, Canada*

[5]*New Zealand Climate Change Research Institute, School of Geography, Environment and Earth Sciences, Victoria University of Wellington, New Zealand*

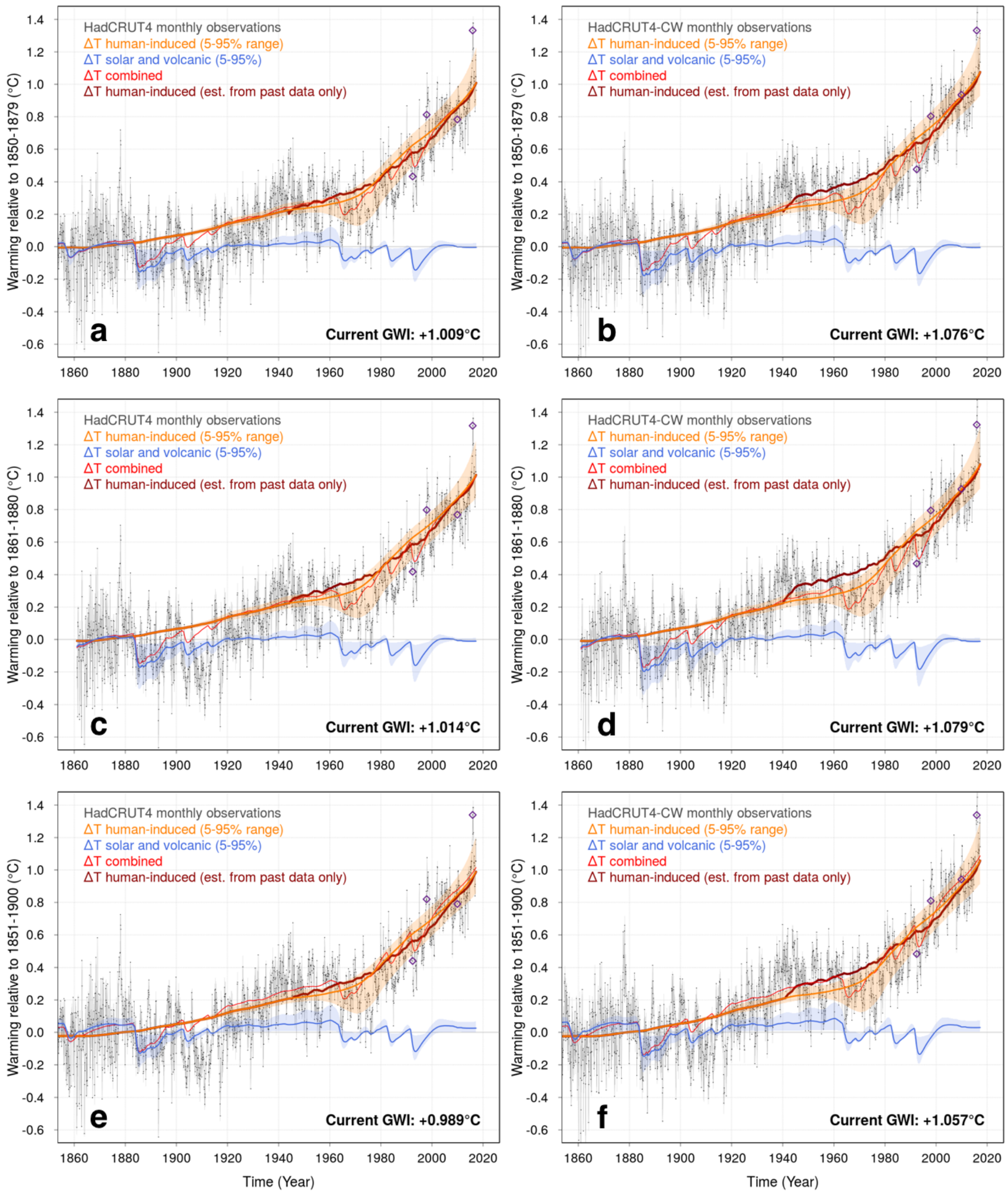

Figure S1: Global Warming Index from Jan 1851/61 to May 2017. (a) Using HadCRUT4 GMST data (full time period of what is shown in Fig. 1). Blue diamonds are the COP months as highlighted in Fig. 1. The reference period is 1850-1879. (b) As (a) but using HadCRUT4-CW GMST data. (c) Same as (a) but with a different reference period of 1861-1880 for pre-industrial conditions. (d) Same as (b), but with the same reference period for pre-industrial conditions as in (c). (e) Same as (a) but for the 1851-1900 reference period. (f) Same as (b) but with same reference period as in (e). The current anthropogenic warming level (May 2017) is shown at the bottom right.

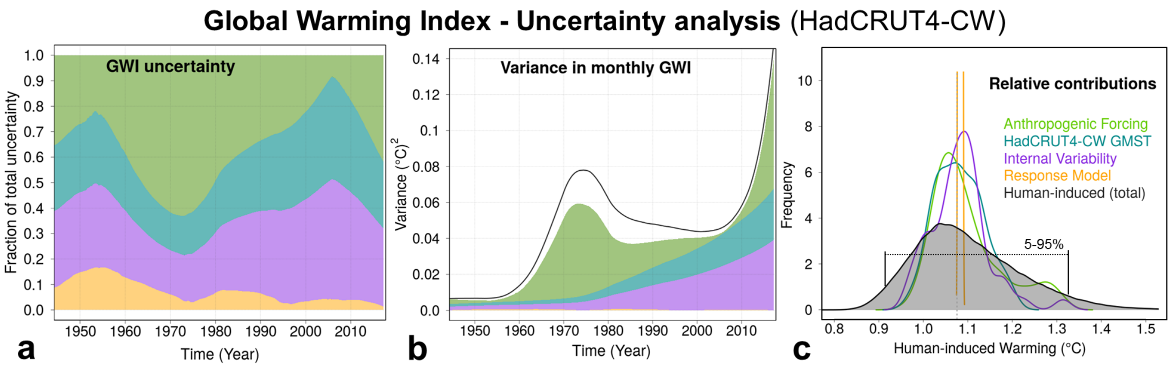

Figure S2: GWI uncertainty analysis. As Fig. 2, but for HadCRUT4-CW.

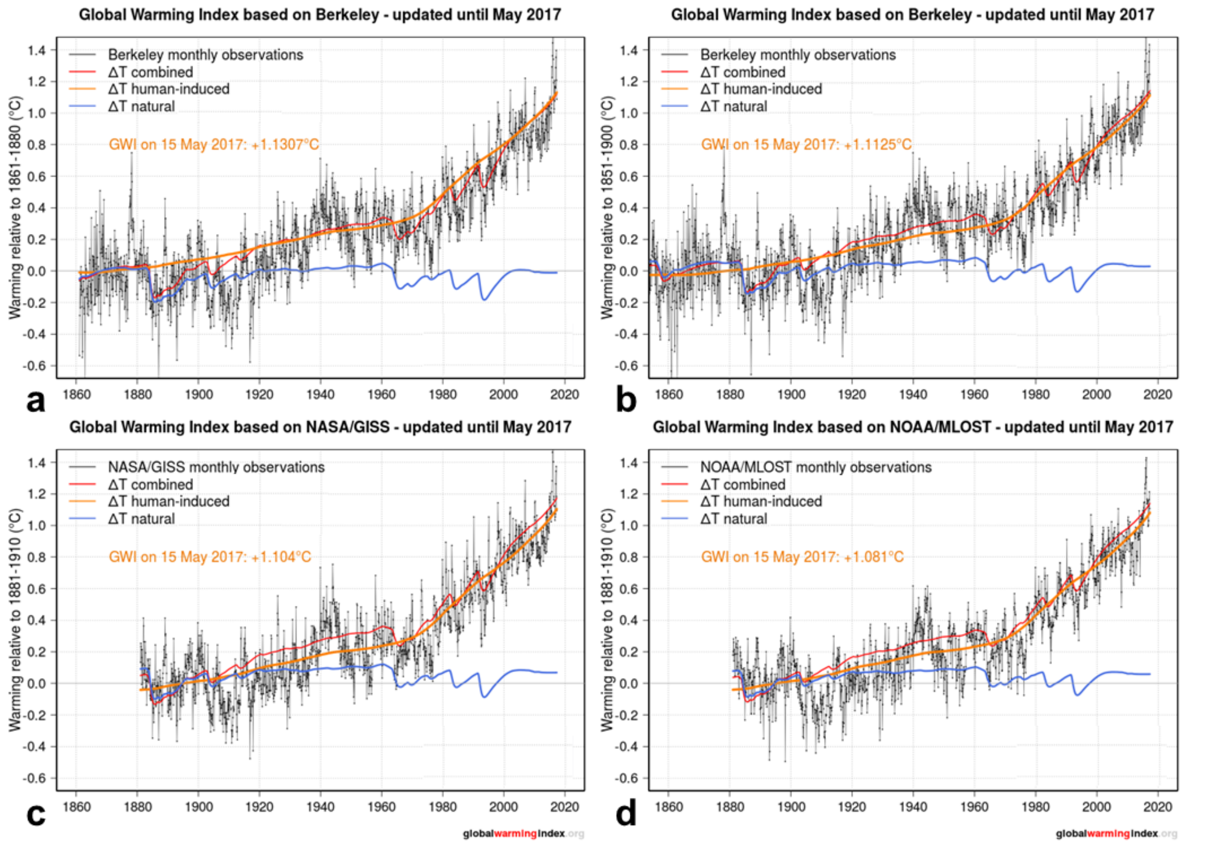

Figure S3: Global Warming Index from Jan 1861/80 to May 2017. As Fig. S1, but for Berkeley Earth, both for (a) the 1861-80 and (b) the 1851-1900 pre-industrial reference period, (c) NASA/GISS and (d) NOAA/MLOST. The latter two are only available after 1881, i.e. the reference period has been shifted to the first 30 years of the record (1881-1910). The associated GWI value for May 2017 is provided in orange. Note the cold anomalies during that period primarily caused by the Krakatoa eruption, leading to a significant natural contribution (blue) to the combined temperature change. Note also that both NOAA and GISS use the GHCNv3 database to estimate their land data.
